# Triple Tick Attack

**DOI:** 10.7759/cureus.4064

**Published:** 2019-02-13

**Authors:** Manish Kumar, Aniket Sharma, Prashant Grover

**Affiliations:** 1 Internal Medicine, University of Connecticut Health Center, Farmington, USA; 2 Internal Medicine, Saint Francis Hospital, Hartford, USA

**Keywords:** ticks, lyme disease, babesia, anaplasma, erythrocytapheresis

## Abstract

Tick-borne diseases are frequently seen in tick-inhabited areas. Lyme disease is the most common tick-borne illness. However, patients with co-infections can present with nonspecific symptoms, which can make the diagnosis far more challenging. We present a case of triple infection with babesiosis, Lyme disease, and anaplasmosis treated with antibiotics and red blood cell (RBC) exchange (erythrocytapheresis). A 74-year-old, avid female gardener presented with one week of progressive dyspnea, cough with mucoid expectoration, and fatigue. On presentation, she was afebrile, hypotensive, and tachycardic. General examination was significant for altered mental status, dyspnea, pallor, and peripheral edema. Lung examination was remarkable for bibasilar crackles. Pertinent laboratory findings were significant for hemolytic anemia and thrombocytopenia. A peripheral blood smear revealed the presence of intracytoplasmic parasites consistent with Babesia. The patient was started on azithromycin and atovaquone. Doxycycline was added empirically for Lyme disease, which was later confirmed by serology. In addition, Anaplasma titers were also positive. Further investigation revealed that the parasitic load was 9.04%, and RBC exchange (erythrocytapheresis) was performed for severe babesiosis. Repeat laboratory tests demonstrated an inadequate reduction in parasitic load (6.54%), requiring a second round of RBC exchange. Antimicrobials were changed to clindamycin, quinine, and doxycycline for a total of 14 days. There was an improvement in the patient’s anemia and thrombocytopenia along with clinical improvement.

## Introduction

Tick-borne diseases are frequently seen in tick-inhabited areas. Lyme disease is the most common tick-borne illness. However, at times, patients may have co-infections leading to nonspecific symptoms, which can complicate the diagnosis. Patients presenting with an atypical clinical picture of a single pathogen or a lack of improvement with antibiotics after 48 hours require further testing for the presence of other infections. A delay in the diagnosis can lead to an increased risk of complications and disease duration.

## Case presentation

A 74-year-old, avid female gardener and active smoker with a past medical history notable for chronic obstructive pulmonary disease (COPD) and hypertension presented to the hospital with one week of progressively worsening New York Heart Association (NYHA) class III dyspnea and fatigue. She endorsed associated cough productive of yellowish mucoid sputum. She denied chest pain/discomfort, palpitations, pre-syncope, syncope, orthopnea, or paroxysmal nocturnal dyspnea (PND). There was no history of fever, arthralgia, myalgia, or rashes.

In the emergency department, the patient was afebrile, blood pressure was 85/49 mm Hg, heart rate was 150 per minute, and respiratory rate was 22 per minute, with oxygen saturation of 94% on six liters of oxygen via a nasal cannula. The patient appeared lethargic and was using accessory muscles for respiration. General examination showed pallor. The oral mucosa was dry, with a thickly coated tongue. The neck veins were flat. Heart examination revealed a fast, irregular heart rate, variable first heart sound, and normal second heart sound without any murmurs or gallops. Lung examination revealed bilateral mid to late inspiratory crackles. The abdomen was soft, distended, non-tender, with normal bowel sounds. Extremities were noted to be cold, with 1+ pitting edema and normal capillary refill time.

**Figure 1:**
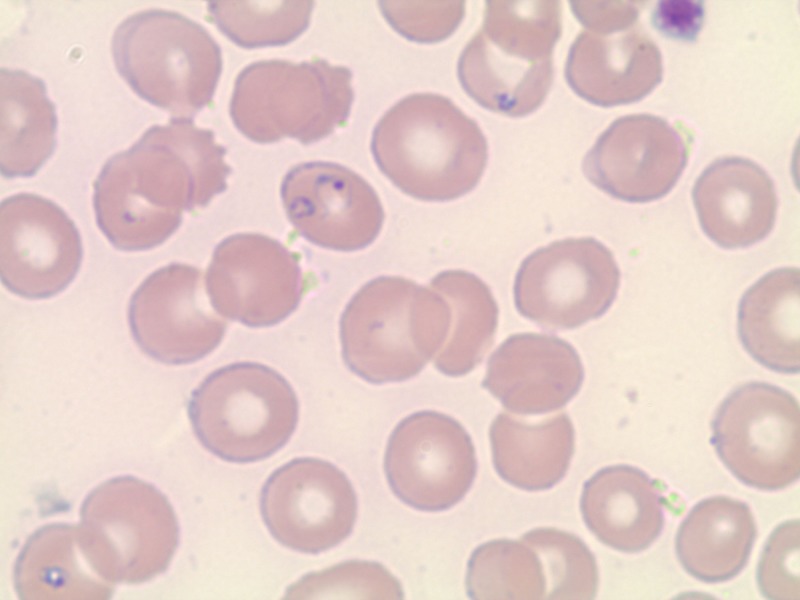
Peripheral blood smear showing numerous intracellular organisms (intra-erythrocytic rings) pathognomonic of babesiosis; also note the reduced number of platelets

Routine laboratory investigations revealed a white blood cell (WBC) count of 7.5 (4.0-10.5 k/uL), hemoglobin of 9.9 (12.5-16 g/dL), and hematocrit of 32.3 (37-47%). Her baseline hemoglobin concentration was around 15 g/dL. Mean corpuscular volume (MCV) was 101.9 (78-100 fL) with elevated mean cell hemoglobin (MCH) of 34 (25-33 pg) and normal mean cell hemoglobin concentration (MCHC) of 33.4 (32-36 g/dL). Platelet count was 34 (150-450 K/uL), with elevated mean platelet volume (MPV) of 12.4 (7.4-11.4 fL). Peripheral blood smear showed intracytoplasmic parasites suspicious for Babesia along with reduced platelets (Figure 1). The parasitic level was found to be at 9.04%. Lactate dehydrogenase (LDH) was 1544 U/L (125-220 U/L), haptoglobin was <6 mg/dL (27-139 mg/dL), total bilirubin was 5.4 mg/dL (0.3-1.0 mg/dL), with a direct fraction of 3.5 mg/dL (0.0-0.2 mg/dL). Aspartate aminotransferase (AST) was 202 U/L (5-40 U/L), alanine aminotransferase (ALT) was 90 U/L (7-52 U/L), with albumin of 2.3 g/dL (3.5-5.0 g/dL). Blood urea nitrogen (BUN) was 51 mg/dL (7-17 mg/dL) with a normal creatinine of 0.8 mg/dL, sodium 129 (135-145 mmol/L), potassium 4 (3.5-5.1 mmol/L), chloride 103 (98-107 mmol/L), serum bicarbonate 19 (24-32 mmol/L), and calcium 7 (8.4-102 mg/dL).

The chest X-ray was remarkable for cardiomegaly with a small right pleural effusion and small airspace opacity within the right lower lobe concerning for consolidation or segmental atelectasis. Given her presentation, she was started on intravenous fluids along with empiric antibiotic coverage for community-acquired pneumonia with ceftriaxone and azithromycin. As the peripheral smear was remarkable for Babesia, she was started on atovaquone and doxycycline for a possible co-infection with Lyme. Blood titers for Anaplasma and Lyme were sent. Positive pressure ventilation was started to help with respiratory distress. She received intravenous fluids; however, she continued to remain hypotensive and required norepinephrine for hemodynamic support. Given the severity of her disease and parasitic load, a decision was made to do erythrocytapheresis.

Following erythrocytapheresis, the parasitic load decreased to 5.54%. The patient remained in shock and clindamycin was added to the regimen. Her renal function worsened, which was believed to be secondary to pigment-induced nephropathy from hemolysis. Due to the inadequate response and development of acute kidney injury, repeat erythrocytapheresis was performed, resulting in a reduction in parasite load to 1.75%. At this point, Lyme immunoglobulin M (IgM) immunoblot was found to be positive, with negative immunoglobulin G (IgG). Anaplasma titers also showed recent/current infection, with IgG >1:1024 (reference <1:64) and IgM 1:80 (reference <1:20). Atovaquone and azithromycin were discontinued and quinine was added. Her hemodynamic status improved and she was titrated off of vasopressor support along with an improvement in respiratory status. Her complete blood count and renal and hepatic function normalized after 14 days of antimicrobial therapy.

## Discussion

Lyme borreliosis (LB) is the most common tick-borne disease in the United States. The causative agent is Borrelia burgdorferi, spread by the tick *Ixodes scapularis*. This tick is known to carry at least seven human pathogens in the United States and Europe, including *Anaplasma phagocytophilum* and *Babesia spp*. [1-2]. It is common for patients to have a co-infection with two pathogens, but the incidence of a three pathogen infection is rare [1,3-4]. Individuals staying in endemic tick areas, such as the North-Eastern United States, are at increased risk.

The typical clinical presentation of Lyme disease includes a characteristic rash known as erythema migrans, intermittent or persistent arthritis, and neurologic manifestations, such as subtle encephalopathy or polyneuropathy. In addition, individuals may have evidence of meningeal irritation, migratory musculoskeletal pain, hepatitis, generalized lymphadenopathy, splenomegaly, sore throat, nonproductive cough, or testicular swelling [5]. Anaplasmosis and babesiosis usually have nonspecific symptoms, such as fever, malaise, myalgia, headache, and chills. Some patients may develop nausea, vomiting, cough, and arthralgia. The clinical manifestations of Babesiosis are mainly due to hemolysis because of the parasite-mediated lysis of erythrocytes. Mild hepato-splenomegaly may be present, however; thrombocytopenia, leukopenia, or anemia are frequently detected. Cases with severe anemia (hemoglobin <10 g/dL) and high parasitic load (>10%) are classified as severe babesiosis and are at increased risk of complications such as acute respiratory failure, disseminated intravascular coagulation, congestive heart failure, and renal failure [3].

A low threshold for suspicion should be held for a co-infection when patients exhibit a presentation that would be atypical for single pathogen exposure. A delay in diagnosis can lead to prolonged disease duration and increases the comorbidities associated with the infectious state [3-4,6].

Various methods are available for diagnosis such as blood smear, polymerase chain reaction (PCR) assay, and serological examinations but none of these methods have 100% sensitivity or specificity. Lyme is diagnosed by two-step testing with enzyme immune-assay (EIA) and Western blot testing; if the EIA is reactive, separate IgM and IgG Western blots are obtained for confirmatory testing. If symptom duration is more than four weeks, IgG Western blot alone is highly sensitive [2]. Anaplasmosis is easily diagnosed by the presence of morulae in neutrophils on a thin smear. PCR and serological testing are also available, which are more sensitive than a thin smear. Titers >1:640 are diagnostic of acute infection with culture as the most sensitive method [7]. A blood smear is the gold standard for the diagnosis of Babesiosis, however, if a patient has a low level of parasitic load, PCR is more sensitive [7-8].

The serological detection of antibodies is not employed routinely for babesiosis. IgG titers of more than 1:1024 suggest an active or recent infection. Positive IgM titers indicate infection and must be accompanied by positive IgG titers for diagnosis [9]. The blood smear may be negative if a patient has a low level of parasitic load. In these cases, if suspicion is high, molecular techniques, such as PCR, should be used to confirm a diagnosis [10]. A multiplex PCR is available for the screening of all three tick-borne pathogens with one PCR reaction [11].

Lyme can be treated with doxycycline, amoxicillin, and cefuroxime, and macrolides are considered second-line agents. A treatment duration of a total of 10 days has been shown to be highly effective in treating both Lyme disease and human granulocytic anaplasmosis [12-13]. In contrast, mild to moderate babesiosis effectively responds to a seven to 10-day course of oral azithromycin and atovaquone, which has been found to be superior to clindamycin and quinine due to the tolerability profile [14]. Various other drug combinations have also been used for treatment such as atovaquone, azithromycin, clindamycin or atovaquone, and clindamycin and artemisinin although none of these regimens have been shown to be superior in comparison to each other [15]. In case of immunocompromised individuals who are at risk of relapsing Babesia, treatment for a total of six weeks is preferred, including a period of two weeks after parasites are no longer visible on a thin smear [15]. Patients with high-grade parasitemia (>10%) with active hemolysis (Hb<10) usually require partial or complete erythropheresis (red blood cell exchange). A 90% reduction should be targeted by exchanging 2.5 times the patient red blood cell volume. Cases with severely complicated babesiosis, such as pulmonary, renal, or hepatic dysfunction, should also receive early erythrocytapheresis, as it may prevent further complications such as disseminated intravascular coagulation (DIC), acute renal failure, or acute respiratory failure [16].

## Conclusions

Lyme disease is the most frequently diagnosed tick-borne disease; however, patients in endemic areas should be screened for a co-infection in cases with nonspecific symptoms and a lack of response to therapy within 48 hours. A 10-day course of doxycycline is usually sufficient for Lyme disease and anaplasmosis while babesiosis usually responds to atovaquone and azithromycin. Erythrocytapharesis should be considered in severe babesiosis, as it may prevent further complications, including severe hemolytic anemia, DIC, respiratory failure, and renal failure.
